# Time changes with feeling of speed: an embodied perspective

**DOI:** 10.3389/fnbot.2014.00014

**Published:** 2014-03-27

**Authors:** Zhijie Zhang, Lina Jia, Weicong Ren

**Affiliations:** ^1^Department of Psychology, Hebei Normal UniversityShijiazhuang, China; ^2^Experimental Psychology, Ludwig Maximilian University of MunichMunich, Germany

**Keywords:** motion, duration, speed words, embodiment, time perception

## Abstract

The speed of moving stimuli can bias duration perception. Here, we investigated whether words describing different speeds influence subjective duration estimation in a temporal bisection task. Duration estimations of two different types of speed words (fast– vs. slow–speed words) were compared. We found that the time bisection point was significantly lower for fast-speed words than for slow-speed words, suggesting that the durations of fast-speed words were overestimated compared to the slow-speed words. In contrast, fast- and slow-speed words did not significantly differ in just noticeable differences and Weber fractions, indicating that the types of speed words did not influence the sensitivity of duration estimation. These results provide new evidence to support the theory of embodied cognition in the context of implicit meaning of a speed word.

## INTRODUCTION

Time is an integral part of our daily life. All our experiences were carried by the river of time. Time perception is often influenced by subjective factors, such as cognitive processes, emotion, or personality (see review, Effron et al., 2006; Wittmann and van Wassenhove, 2009; Nather et al., 2011). Although as a subjective experience, time perception is also influenced by physical characteristics of stimuli, such as movement.

Movement-related factors, such as the moving speed of a stimulus, have been demonstrated to bias time perception (Brown, 1995; Kaneko and Murakami, 2009; Wittmann et al., 2010; Matthews, 2011; Au et al., 2012). Specifically, Brown (1995) found that, in the range of seconds, visual moving stimuli were estimated longer in duration relative to static stimuli, and fast-speed stimuli were perceived as longer than slow-speed stimuli for durations. This phenomenon was also observed in sub-second time range (Kaneko and Murakami, 2009; Au et al., 2012).

Time perception was also biased by embodiment, another movement-related factor (Effron et al., 2006; Nather et al., 2011; Wang and Jiang, 2012). Nather et al. (2011) reported that the estimation on how long a picture of body posture is presented depended on what body posture is in that picture. Such an effect was interpreted in terms of embodied theories of cognition, which assumes that cognition is grounded in body–environment interactions and abstract concepts are represented in the body’s sensorimotor experiences. Embodied simulation involves specific sensory reactions of physical states (e.g., imitation) corresponding to the abstract concepts (e.g., the movement meaning of a body picture) (Barsalou, 2003; Barsalou and Wiemer-Hastings, 2005). In other words, embodiment refers to actual bodily states and to simulations of experience involving perception, action, and introspection in the brain (Niedenthal et al., 2005). One typical example is the imitation of emotional facial expression. When viewing emotional facial expressions, observers often spontaneously imitate them by activating the corresponding facial muscles (Dimberg, 1982). Thus, when participants view and embody stimuli with action meanings, their motor systems are activated, which in turn changes time perception.

Movement effects on subjective duration estimation can be explained within the frame of the internal clock model (e.g., Effron et al., 2006; Kaneko and Murakami, 2009; Matthews, 2011; Nather et al., 2011). According to this model, time information is recorded in an internal clock composed of three parts: a pacemaker that emits pulses at a regular rate, a switch controlling the start and the stop of timing, and an accumulator that receives the pulses transmitted from the switch (Treisman, 1963; Gibbon et al., 1984; Zakay and Block, 1996). Time perception is associated with the number of pulses accumulated in the accumulator. Thus, movements can change duration estimation possibly by mediating the internal pacemaker speed with more pulses emitted in the unit time for a moving stimulus than a stationary stimulus (Kaneko and Murakami, 2009). One explanation to this increased clock processing speed is the arousal level (Effron et al., 2006). As for the role of embodiment on time perception, researchers assume that embodiment of stimuli related to movement may trigger high arousal, which in turn speeds up the clock processing (Effron et al., 2006). It has been revealed that imitation of emotional stimuli can modulate arousal levels (Vaughan and Lanzetta, 1981; Zuckerman et al., 1981). Thus, embodiment might affect time perception by mediating arousal. It is also possible that moving stimuli capture more attention due to their salience. Attention controls switch latency and the closure state of switch. When more attention is given to the processing of moving stimuli, more temporal pulses would be accumulated and the perceived duration is extended (Yarrow et al., 2004; Au et al., 2012).

The present study aimed to investigate whether the implicit motion information implied in speed words would affect duration perception. Based on the theory of embodied cognition, we predicted that the duration of fast-speed words would be overestimated compared with slow-speed words.

## MATERIALS AND METHODS

### PARTICIPANTS

Thirty-two volunteers (mean age = 23.47 years, SD = 2.14, 14 male) from Hebei Normal University participated in this experiment. All participants were right-handed and had normal or corrected-to-normal vision. All participants were given written informed consent approved by the ethical committee of Hebei Normal University.

### MATERIALS

Visual stimuli were presented on a 17-inch CRT color monitor with a screen resolution of 800 pixels × 600 pixels and a refresh rate of 75 Hz. The viewing distance was 60 cm and constant throughout the experiment. Visual stimuli were white and shown on black background. Standard visual stimuli were squares (2 cm × 2 cm). Comparison stimuli involved two types of speed words (36-point Courier New) in Chinese words: five fast-speed words (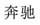-gallop, 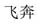-run like the wind, 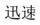-rapid, 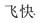-fast like flying, and 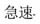-hurried at a great speed ) and five slow-speed words (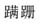-limp, 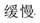-slow, 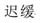-sluggish, 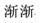-gradual, and 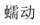-creep). These words were selected based on a pilot experiment, in which 100 undergraduate students were asked to come up with Chinese words indicating fast or slow speed. The frequencies of these words were counted. The five words with the highest frequency within each category (fast and slow) were selected as the final target words.

### PROCEDURE

We utilized a within-subjects design and a temporal bisection task to examine whether the implicit motion information implied in speed words would bias duration perception. Two main factors were: word type (two levels: fast and slow) and comparison duration (five levels: 400, 600, 800, 1000, and 1200 ms). The temporal bisection task included a training session and a test session. In the training session, the visual standard stimuli (i.e., white squares) were presented for either a short (400 ms) or a long duration (1200 ms) with each duration presented for five times. The order of the standard visual stimuli was randomized. Participants were asked to discriminate these two durations. All participants reached 100% accuracy in their performance before starting the test session.

The participants initiated the test session by pressing the “space” button. For the test session, a target word randomly selected from five fast-speed and five slow-speed words was presented for a given length of duration. After presenting the target word, a red exclamation mark (“!”) appeared on the screen prompting the participant to compare the presented duration of the target word with one of the two standard durations presented during the training session. If it was closer to the short standard duration (400 ms), then the participants were required to press key “D”. If it was closer to the long standard duration (1200 ms), then they were required to press key “K”. Participants were asked to fix their eyes on the center of the screen throughout the experiment. The inter-trial interval (ITI) was 1 s. A total of 50 trials (i.e., 10 target words x 5 duration lengths) were pseudo-randomly separated into five blocks. The experimental programs were developed using E-prime 2.0 (Schneider et al., 2002).

## RESULTS

**FIGURE 1:**
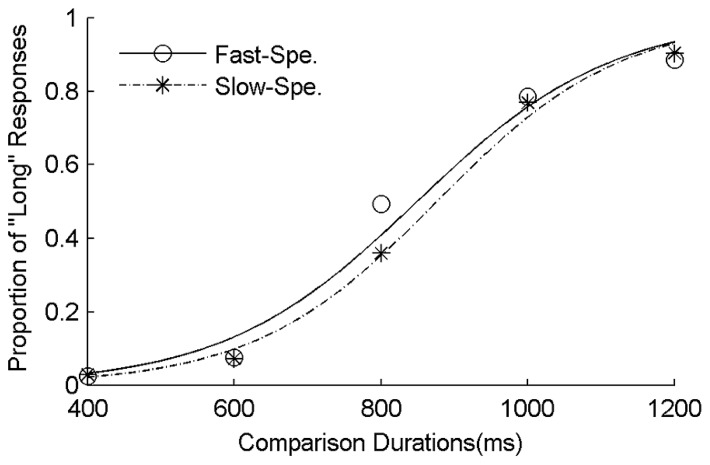
**Mean proportions of “Long” responses plotted against probe durations and fitted psychometric functions for the fast-speed words and slow-speed words conditions.** The dashed curve and asterisks represented the slow-speed word condition, the solid curve and circles the fast-speed word condition.

The proportions of “long” responses were calculated for each condition and each subject. This measure was then fitted by logistic functions against the comparison durations. A higher proportion of “long” responses indicate that participants are more frequent to judge the comparative duration as closer to the long standard duration. **Figure 1** shows the average psychometric curves for the fast-speed word and slow-speed word condition, separately. The temporal bisection point (TBP) was the 50% point of the logistic function (Treutwein and Strasburger, 1999). A lower TBP means a longer perceived duration and a higher TBP means a shorter perceived duration. The just noticeable difference (JND) of the temporal bisection was the half difference in duration between the 25% and 75% points of the logistic function (Shi et al., 2008; Vroomen and Keetels, 2010). JND is the indicator of the sensitivity of the temporal bisection task. In addition, we measured the Weber fraction, calculated as the ratio of JND/TBP.

**FIGURE 2:**
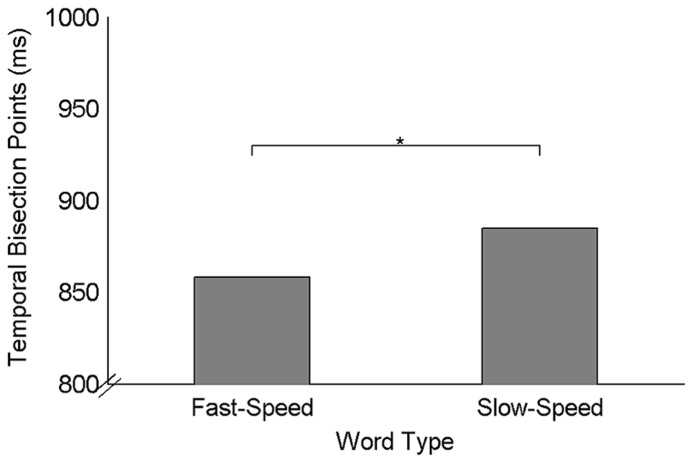
**Mean temporal bisection points for two types of speed words.** *p < 0.05.

A 2 (word type) × 5 (comparison duration) repeated-measures ANOVA was performed on the proportions of “long” response. The main effects of word type [*F*(1,31) = 5.84, *p * < 0.05, $\eta_{p}^{2}$ = 0.16] and comparison duration [*F*(4,124) = 362.90, *p * < 0.05, $\eta_{p}^{2}$ = 0.92], as well as their interaction [*F*(4,124) = 6.05, *p * < 0.05, $\eta_{p}^{2}$ = 0.16], were all significant. Following up the interaction effect, paired-sample *t*-tests were performed to compare the proportion of “long” responses computed for fast-speed words with that computed for slow-speed words in each comparison duration. Results indicated that the fast-speed words were perceived longer than slow-speed words only for the 800 ms comparison duration, but not for other durations. Furthermore, three one-way repeated-measures ANOVAs with word type as a factor were performed on TBPs, JNDs, and Weber fractions, respectively. Results of TBPs indicated that the type of speed words significantly influenced duration estimation [*F*(1,31) = 5.36, *p *< 0.05, $\eta_{p}^{2}$ = 0.15], with the duration of the fast-speed words being judged longer (TBPs: 858 ms ± 17 ms) than the duration of the slow-speed words (TBPs: 885 ms ± 19 ms; **Figure 2**). Results of JNDs indicated that the type of speed words did not affect the sensitivity of temporal judgment [*F*(1,31) = 0.48, *p *= 0.50, $\eta_{p}^{2}$ = 0.02]. Results of Weber fractions indicated that the main effect of word type was not significant [*F*(1,31) = 0.7, *p *= 0.41, $\eta_{p}^{2}$ = 0.02], suggesting that the task difficulties were similar between two speed conditions.

## DISCUSSION

The effect of implicit information implied in speed words on time perception was compared between two conditions: slow-speed and fast-speed. The key finding is that participants classified the duration of fast-speed words as “long” standard duration more frequently relative to that of slow-speed words. In other words, the duration of fast-speed words was perceived as longer than slow-speed words. This finding is consistent with previous studies (Brown, 1995; Kaneko and Murakami, 2009; Matthews, 2011) and extended previous work by showing that implicit meaning of speed indicated by words expanded subjective duration.

Compared with previous research using visual motion or display to simulate physical movement (Brown, 1995; Kaneko and Murakami, 2009; Matthews, 2011; Au et al., 2012), the influence of speed words on time perception may indicate a social interaction between external social signals (linguistic) and perception. Our internal timing mechanism may adjust its speed to adapt to the speed implied by linguistic. Thus, as predicted by embodiment theory, the biased subjective time induced by the speed words may be a result of the automatic synchronization of our bodily rhythm with social environment (see review Droit-Volet and Gil, 2009). For example, viewing words (old vs. neutral) before actions modulated the actual action of participants (Bargh et al., 1996). Slower actions were observed when participants were presented with the old-related words (e.g., lonely) compared to being presented with neutral words (e.g., clean). As for the relationship between imitation and time perception, Chambon et al. (2008) found that durations of elderly faces were judged shorter than young faces.

Indeed, a mirror neuron circuit has been identified in the neuroimaging literature to produce motor mimicry in response to perceived actions (Gallese et al., 2004; Keysers et al., 2004; Lloyd et al., 2006; Cattaneo and Rizzolatti, 2009). One key region of this circuit is the primary somatosensory cortex, which exhibited greater activation when viewing a rubber hand being touched by a sharp (painful) compared to a blunt (non-painful) stimulus (Lloyd et al., 2006). These results suggested that the mirror neurons may underpin the body adjustment in adaptation to the action meaning indicated by external stimuli.

No variation mechanism (clock speed, switch, and memory) in the internal clock seem to be mediated directly by embodiment and then bias time perception. Alternatively, arousal state associated with embodied simulation may change the speed of the pacemaker in the internal clock (Effron et al., 2006; Nather et al., 2011). For example, Effron et al. (2006) reported that the effect of emotional facial expressions on temporal estimation was modulated by the participants’ imitation. Compared to the free imitation condition, duration expansion was diminished when a participant’s imitation was inhibited by holding a pen in the mouth. This is probably because automatic free imitation might lead to higher arousal states as participants also experience angry or happy feelings (Droit-Volet and Meck, 2007). Consequently, the clock is sped up and the subjective duration is expanded. In contrast, when the imitation is inhibited, a high arousal level is not triggered, thus the subjective duration is not expanded. Postures with more movements may trigger a higher arousal level and participants’ body states can be activated more by imitating those postures (Nather et al., 2011). As a result, a longer subjective duration was perceived. However, JND was not significantly different between slow- and fast-speed words in the current study, suggesting that arousal did not contribute to the current results (Nather et al., 2011).

Recently, two other theories were proposed to account for the role of embodiment on time perception: structure model of awareness and coding efficiency theory. The awareness model suggested that time perception is provided by the integration of self-referential moments across time (Craig, 2009). When an emotional time event is given, these salient moments run fast and the number of global emotional moments increases. As a result, our subjective time is expanded. This model emphasizes the influence of dynamic bodily states on subjective time. Consistent with this model, Wittmann et al. (2010) reported that looming stimuli simulating a movement toward the observer were perceived longer in duration than when they were receding (moving away) or when they were static. These behavioral results were further supported by fMRI studies demonstrating that midline structures implicated in self-processing were involved in this phenomenon. Compared to slow-speed words, fast-speed words may trigger more movements, and might prompt our body to embody more quickly. The key point of the awareness model is that body states modulate time perception. Therefore, fast-speed words might trigger a high rate of integration of sentient self across time, which filled moments quickly. In contrast, the speed of moments in the slow-speed condition ran slowly to imitate the slow speed indicated by words. Consequently, subjective duration was lengthened for fast-speed words relative to slow-speed words.

Coding efficiency theory suggests that subjective duration is a result of coding efficiency (Eagleman and Pariyadath, 2009). The experience of duration is a signature of the amount of energy expended in representing a stimulus. For example, the visual information is sped up during full motor preparation (Hagura et al., 2012). In this regard, it seems that stimuli involving motor components would be processed by more energy relative to baseline. In the present study, fast-speed words indicate more movements than slow-speed words. Thus, when presenting fast-speed words (e.g., 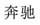-gallop) to participants, more energy might be expended for processing these words during embodiment (i.e., imitation) compared to slow-speed ones (e.g., 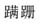-limp).

To summarize, we found that the perception of duration is biased by speed words. Durations of fast-speed words were expanded compared to slow-speed words. Our results support the important role of embodiment in time perception, which is possibly due to self-referential processing or an increased efficiency in information processing.

## Conflict of Interest Statement

The authors declare that the research was conducted in the absence of any commercial or financial relationships that could be construed as a potential conflict of interest.
